# Enhancing Bioactivity and Conjugation in Green Coffee Bean (*Coffea arabica*) Extract through Cold Plasma Treatment: Insights into Antioxidant Activity and Phenolic–Protein Conjugates

**DOI:** 10.3390/molecules28207066

**Published:** 2023-10-13

**Authors:** Kuntapas Kungsuwan, Choncharoen Sawangrat, Sakaewan Ounjaijean, Supakit Chaipoot, Rewat Phongphisutthinant, Pairote Wiriyacharee

**Affiliations:** 1 Division of Product Development Technology, Faculty of Agro-Industry, Chiang Mai University, Chiang Mai 50100, Thailand; kuntapas_k@cmu.ac.th; 2 Department of Industrial Engineering, Faculty of Engineering, Chiang Mai University, Chiang Mai 50200, Thailand; choncharoen@step.cmu.ac.th; 3 Agriculture and Bio Plasma Technology Center (ABPlas), Thai Korean Research Collaboration Center (TKRCC), Science and Technology Park, Chiang Mai University, Chiang Mai 50100, Thailand; 4 School of Health Sciences Research, Research Institute for Health Sciences, Chiang Mai University, Chiang Mai 50200, Thailand; sakaewan.o@cmu.ac.th; 5 Multidisciplinary Research Institute (MDRI), Chiang Mai University, Chiang Mai 50200, Thailand; supakit.ch@cmu.ac.th; 6 Center of Excellent in Microbial Diversity and Sustainable Utilization, Faculty of Science, Chiang Mai University, Chiang Mai 50200, Thailand

**Keywords:** cold plasma, green coffee bean extract, antioxidants, conjugates, bioactive compounds

## Abstract

Cold plasma technology is gaining attention as a promising approach to enhancing the bioactivity of plant extracts. However, its impact on green coffee bean extracts (GCBEs) still needs to be explored. In this study, an innovative underwater plasma jet system was employed to investigate the effects of cold plasma on *Coffea arabica* GCBEs, focusing on the conjugation reflected by the change in composition and bioactivity. The DPPH radical scavenging antioxidant activity exhibited a gradual increase with plasma treatment up to 35 min, followed by a decline. Remarkably, at 35 min, the plasma treatment resulted in a significant 66% increase in the DPPH radical scavenging activity of the GCBE. The total phenolic compound content also displayed a similar increasing trend to the DPPH radical scavenging activity. However, the phenolic profile analysis indicated a significant decrease in chlorogenic acids and caffeine. Furthermore, the chemical composition analysis revealed a decrease in free amino acids, while sucrose remained unchanged. Additionally, the SDS-PAGE results suggested a slight increase in protein size. The observed enhancement in antioxidant activity, despite the reduction in the two major antioxidants in the GCBE, along with the increase in protein size, might suggest the occurrence of conjugation processes induced by plasma, particularly involving proteins and phenolic compounds. Notably, the plasma treatment exhibited no adverse effects on the extract’s safety, as confirmed by the MTT assay. These findings indicate that cold plasma treatment holds significant promise in improving the functional properties of GCBE while ensuring its safety. Incorporating cold plasma technology into the processing of natural extracts may offer exciting opportunities for developing novel and potent antioxidant-rich products.

## 1. Introduction

Green coffee bean extract, derived from unroasted coffee beans, is well known for its bioactive components that contribute to potential health benefits. Major compounds found in green coffee beans include chlorogenic acids, caffeine, and other polyphenols [1,2]. These bioactive constituents have been closely linked to a reduction in oxidative stress and inflammation within the body, thereby playing a role in enhancing cardiovascular health [3]. Moreover, GCBEs are often associated with aiding in weight management and the attenuation of diabetes as it has demonstrated the potential to regulate blood sugar levels and stimulate metabolism [4]. Additionally, emerging research hints at the neuroprotective properties of GCBEs, suggesting potential support for cognitive function [5].

Cold plasma, a promising nonthermal technology, has garnered increasing attention in the food industry due to its potential applications. Notably, it has demonstrated efficacy in reducing microbial contamination [6] and inhibiting degradation enzymes [7], thereby extending the shelf life of food products [8]. Moreover, cold plasma can effectively remove harmful chemicals such as pesticides from the product, improving its safety [9,10].

Recent advancements have extended the application of cold plasma to enhance the bioactivity and preservation of plant extracts. Cold plasma generates reactive oxygen and nitrogen species, which induce chemical reactions and modify the composition of treated substances. This technology significantly improves extraction efficiency by breaking down complex molecules and cell walls [11], releasing more bioactive compounds from the plant material. Moreover, it can modify the chemical structures of these compounds, potentially increasing their bioavailability and functionality [12,13].

While existing research predominantly focuses on optimizing the processes before and during extraction using cold plasma [11,14,15], a substantial knowledge gap remains regarding its full impact on modifying bioactive compounds directly in the extract. Further exploration is necessary to uncover the potential benefits of cold plasma treatment in preserving and enhancing the bioactivity of plant extracts.

Conjugation processes in food chemistry are foundational reactions that significantly affect diverse food products’ sensory attributes, color, flavor, and nutritional composition. These processes can involve interactions among phenolic compounds, carbohydrates, and proteins within green coffee beans, potentially leading to compositional changes and heightened bioactivity, thus influencing the final coffee product. One noteworthy instance of such conjugation is the Maillard Reaction. This well-established reaction typically occurs during heating, where amino acids (proteins) interact with reducing sugars (carbohydrates), giving rise to a complex array of flavor compounds and the characteristic browning of food [16]. Concurrently, phenolic compounds can engage in phenolic–protein conjugation, potentially elevating antioxidant capacity and conferring potential health benefits [17]. The application of cold plasma treatment presents an innovative avenue to initiate and modulate these conjugation processes in food. Extensive research underscores how reactive species from plasma can induce depolymerization and oxidation, particularly in proteins, resulting in conjugation and structural modifications that can further enhance the complexity and quality of food products [18,19]. 

Therefore, this research aims to comprehensively investigate the impact of cold plasma treatment on green coffee bean extract (GCBE). With a specific focus on the hypothesis that cold plasma induces chemical conjugation in GCBE, leading to notable changes in composition and bioactivity, this study explores the potential exposures to plasma to enhance the bioactivity of GCBE while emphasizing the critical role of treatment duration. Furthermore, we assess the safety of the process to determine its viability for producing safer and potentially more bioactive GCBE. By delving into these aspects, we hope to shed light on the untapped potential of cold plasma technology in improving green coffee bean extract production.

## 2. Results

### 2.1. Changes in GCBE Composition

The initial phase of the study aimed to assess the impact of plasma treatments on the composition of the GCBE, with a specific focus on variations in protein, sugar, and phenolic compounds. To evaluate changes in protein, we investigated alterations in the content of free amino acids, which could serve as indicators of modifications in smaller protein units. Additionally, we analyzed protein size through SDS-PAGE to understand changes in the overall protein bulk. The differences in sugar content were measured by analyzing the sugar profile to identify any shifts resulting from the plasma treatments. Moreover, changes in phenolic compounds were observed by measuring the total phenolic content (TPC) and the phenolic profile.

The GCBE samples were exposed to plasma treatments for different durations, with untreated GCBE serving as the control and treated samples for 10, 35, and 60 min of plasma exposure, designated as P10, P35, and P60, respectively. Table 1 displays the changes in free amino acids, sucrose, total phenolic content, chlorogenic acid, and caffeine in the GCBE and GCBE exposed to plasma treatments.

The analysis of the free amino acids in the GCBE samples revealed significant changes following plasma treatment. The total free amino acid content decreased from 392.16 ± 1.62 mg/L in the control sample to a minimum of 316.00 ± 15.64 mg/L at P60. Among the 17 amino acids analyzed, varying degrees of change were observed. The results are summarized in Table 2. Interestingly, several amino acids showed no significant changes with plasma treatment, including aspartate, threonine, serine, glutamic acid, proline, alanine, and cysteine. This result suggests that these amino acids are relatively stable and not susceptible to oxidation by reactive oxygen species (ROS) generated during plasma treatment. In contrast, higher plasma treatment times resulted in reduced valine, histidine, and lysine levels, which contain reactive functional groups prone to oxidation. Conversely, slight increases were observed in glycine, methionine, isoleucine, leucine, tyrosine, phenylalanine, and arginine levels.

The profiles of four sugars, glucose, fructose, sucrose and galactose, were determined for all samples. The results revealed that only sucrose was present in the GCBE samples. The sucrose levels remained relatively unchanged following plasma treatment, suggesting its stability.

TPC, analyzed using the Folin–Ciocalteu method, exhibited an increasing trend following plasma treatment for 10 min (P10) and 35 min (P35), reaching a maximum of 7.63 ± 0.26 g GAE/L at P35. However, TPC decreased at P60.

Conversely, the analysis of chlorogenic acid and caffeine, which are the major antioxidants in green coffee bean extract, showed distinct patterns. The chlorogenic acid content exhibited a gradual decline during plasma treatment, decreasing from 5.10 ± 0.01 to a minimum of 4.17 ± 0.77 at P60. In contrast, the caffeine content showed a slight decreasing trend with increasing plasma treatment time, although it did not reach statistical significance.

Further confirmation of the reduction in antioxidants in GCBE due to plasma treatment was obtained through a phenolic profile analysis (Table 3) of the control and P35 samples. Among the 18 phenolic compounds examined, 6 were identified in GCBE: chlorogenic acid, caffeine, vanillic acid, vanillin, p-coumaric acid, and myricetin. Plasma treatment for 35 min significantly reduced the levels of chlorogenic acid and caffeine.

### 2.2. Change in Antioxidant Activity due to Plasma Treatment

Antioxidant assays are crucial in evaluating the potential health benefits of various substances, including natural extracts. In this study, we investigated the effect of plasma treatment on the antioxidant activity of GCBE using a series of assays.

The primary assay used was the DPPH radical scavenging assay, which provides valuable insights into the extract’s ability to neutralize free radicals. Additionally, we employed the ABTS and FRAP assays to explore different antioxidant mechanisms. To gain a deeper understanding of the extract’s antioxidant potential at the cellular level, we conducted the cell antioxidant assay (CAA).

**Figure 1:**
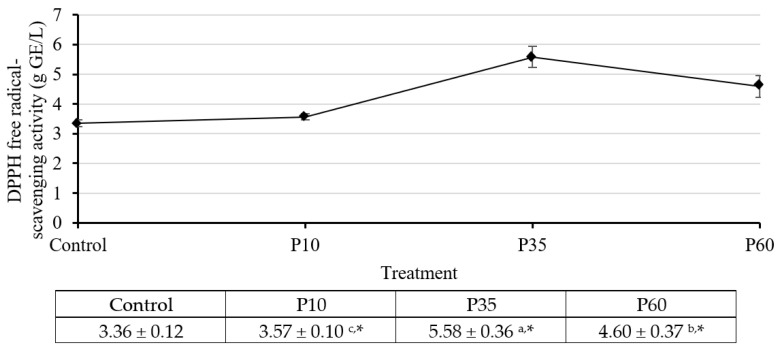
The DPPH radical scavenging activities assay results of GCBE (control) and GCBEs treated with plasma for 10 min (P10), 35 min (P35), and 60 min (P60). ^a,b,c^ Values with different letters within the same row differ significantly with a *p*-value ≤ 0.05, as indicated by Duncan’s new multiple range test. * Values differ significantly from the control with a *p*-value ≤ 0.05, as indicated by Student’s *t*-test.

Firstly, we evaluated the effect of plasma treatment using the DPPH radical scavenging assay, and the results are presented in Figure 1. We observed a significant improvement in antioxidant activity with increasing plasma treatment duration. The maximum activity was observed at P35, with a value of 5.58 ± 0.36 g GAE/L. However, at P60, the activity decreased, indicating a time-dependent effect. Based on these findings, we chose the plasma treatment at 35 min to further evaluate antioxidant activity in other assays. These additional assays included the ABTS radical scavenging, FRAP, and cell antioxidant (CAA) assay.

As shown in Table 4, unlike the DPPH assay, the ABTS and FRAP assays did not show significant differences between the control and plasma-treated samples. The ABTS assay demonstrated a slightly increasing trend, while the FRAP assay showed a decreasing trend.

**Figure 2:**
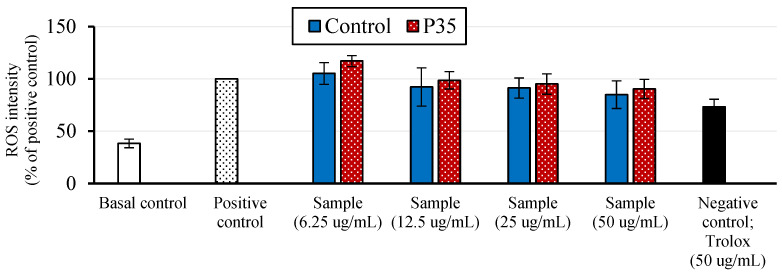
Cellular antioxidant activity (CAA) assay results of GCBE (control) and GCBE treated with plasma for 35 min (P35).

The cell antioxidant assay (CAA) was then conducted to further understand the extract’s antioxidant potential at the cellular level. According to Figure 2, at a concentration of 6.3 mg/L, the control and P35 samples induced ROS production in the cells. However, the extracts exhibited concentration-dependent increases in cell antioxidant activity at higher concentrations. There was a slight decreasing trend in ROS intensity observed with plasma treatment when we compared the control and P35 samples; however, this decrease did not reach statistical significance. In addition, it is worth noting that the antioxidant efficacy of GCBEs was lower than that of Trolox, a known synthetic antioxidant.

### 2.3. Effect of Plasma Treatment on Protein Size 

**Figure 3:**
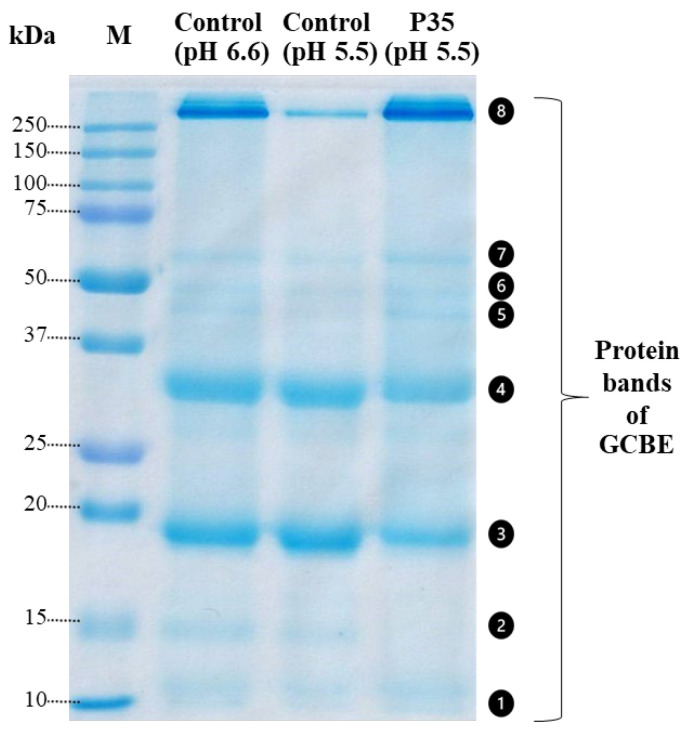
SDS-PAGE gel of GCBE (control) and GCBE treated with plasma for 35 min (P35).

In Figure 3, the SDS-PAGE analysis of the GCBE samples treated with plasma for 35 min (P35) and the control samples at two different pH values (pH 6.6 and pH 5.5) is presented. The purpose of performing the SDS-PAGE at two pH levels was to account for the potential effect of pH on the protein size.

The GCBE samples exhibited eight protein bands of varying sizes. However, noticeable differences were observed between the two samples. In the P35 sample, band No. 2 disappeared, and bands No. 3 and No. 4 exhibited a slight fading, suggesting a reduction in the number of proteins in these bands due to plasma treatment.

Conversely, an increase in the intensity of a protein band above 250 kDa (band No. 8) was observed, indicating either an increase in the size of proteins or an increase in the amount of proteins larger than 250 kDa. These findings suggest that plasma treatment may have induced changes in the protein composition of the GCBE sample, leading to alterations in protein size and abundance.

### 2.4. Effect of Plasma Treatment on GCBE Chemical Composition Using Fourier-Transform Infrared (FT-IR) Spectroscopy

FT-IR spectroscopy was employed to investigate the effect of plasma treatment on the chemical composition of GCBE. The FT-IR spectra of the untreated and plasma-treated extracts were analyzed, and specific wavenumber shifts were observed. 

**Figure 4:**
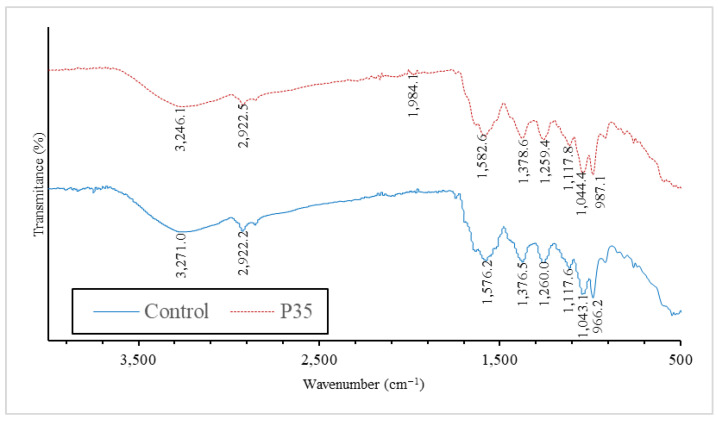
FT-IR spectrum of GCBE (control) and GCBE treated with plasma for 35 min (P35).

The FT-IR results demonstrate that the plasma treatment induced notable changes in the infrared absorption spectra of the GCBE. Specific wavenumber shifts were observed, indicating alterations in the functional groups and chemical bonds present in the extract. Figure 4 shows the FT-IR spectrum of the GCBE compared with the GCBE treated with plasma (P35).

The shift observed at 1582.6 cm^−1^ (from 1576.2 cm^−1^) suggests changes in the C=C stretching vibrations, which are typically associated with aromatic compounds. This shift indicates modifications in the aromatic structure of GCBE, possibly due to the generation of new functional groups or changes in existing double bond configurations after plasma treatment.

The shift observed at 1259.4 cm^−1^ (from 1260 cm^−1^) may indicate variations in the C-O-C stretching vibrations commonly observed in ethers and esters. This shift implies that plasma treatment could have led to ether or ester linkage alterations.

The shift observed at 1378.6 cm^−1^ (from 1376.5 cm^−1^) may be associated with changes in the C-H bending vibrations, suggesting potential modifications in aliphatic hydrocarbon chains present in the extract. This shift could indicate alterations in the carbon–hydrogen bond arrangements due to plasma treatment.

The shift observed at 3246.1 cm^−1^ (from 3271 cm^−1^) implies changes in the O-H stretching vibrations, which are characteristic of hydroxyl groups. 

### 2.5. Effect of Plasma Treatment on Antimicrobial Activity 

**Figure 5:**
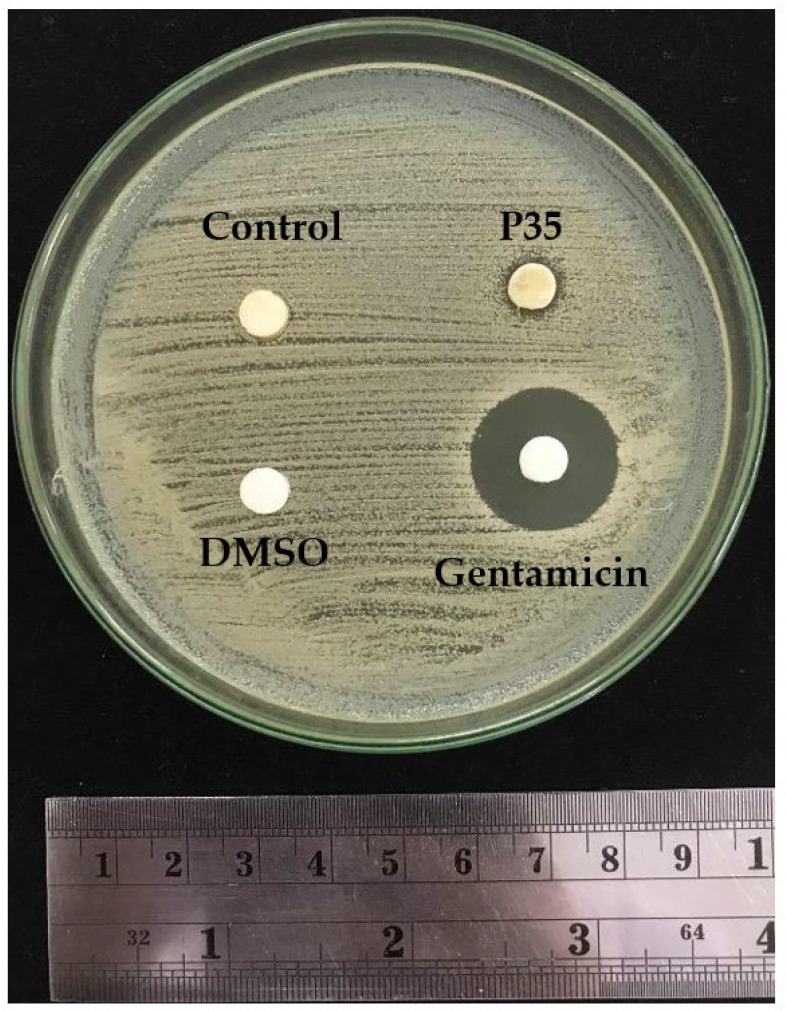
Disk diffusion plate of GCBE (control) and GCBE treated with plasma for 35 min (P35) showing inhibition zone against *S. aureus*.

Agar diffusion was employed to evaluate the antimicrobial activity of the GCBE samples. DMSO served as the negative control, while gentamicin was utilized as a positive control. Figure 5 displays the antimicrobial results of the GCBE (control) and plasma-treated GCBE at 35 min (P35). The agar diffusion assay revealed that the GCBE exhibited no antimicrobial activity against *S. aureus*. However, plasma treatment for 35 min showed a modest improvement in antimicrobial activity, indicated by a subtle decrease in the bacterial colony around the sample. Although the affected area was small and not substantial enough to be classified as a clear inhibition zone against *S. aureus*, these results suggest a slight enhancement of the antimicrobial activity of GCBE due to plasma treatment.

### 2.6. Effect of Plasma Treatment on Toxicity

In our study, we employed HepG2 cells, a widely recognized human hepatocellular carcinoma model, due to their ability to faithfully replicate key aspects of hepatic physiology. This choice was motivated by their relevance to the study of liver-related pathways and their applicability to investigating bioactive compounds present in green coffee bean extract (GCBE). Notably, chlorogenic acid, the principal bioactive compound in GCBE, is proposed to undergo metabolism primarily in the liver and kidneys [20]. Furthermore, our selection aligns with previous research employing HepG2 cells to assess the effects of cold plasma treatment on various food samples [21,22].

**Figure 6:**
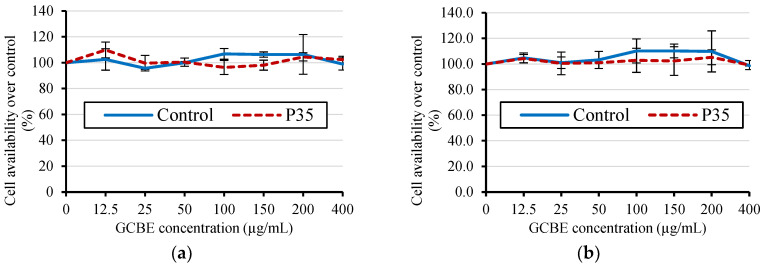
MTT cell viability after treatment with GCBE (control) and GCBE treated with plasma for 35 min (P35): (**a**) HepG2 cells after 24 h; (**b**) HepG2 cells after 48 h.

Figure 6 displays the MTT cell viability results of the GCBE and GCBE treated with plasma for 35 min (P35) in HepG2 cells (liver cancer cells). The MTT assay was used to assess GCBE’s cytotoxicity before and after cold plasma treatment.

Surprisingly, the results showed no significant difference in cell viability between the untreated and plasma-treated groups. Both groups exhibited approximately 100% cell viability, indicating that cold plasma treatment had no significant impact on GCBE’s cytotoxicity.

The consistent cell viability across both groups suggests that the cold plasma treatment, as applied in this study, did not induce an increase in the toxicity of the GCBE. This finding supports the notion that cold plasma treatment is a safe and non-toxic approach for treating GCBEs.

## 3. Discussions

This study comprehensively investigated the effects of cold plasma treatment on green coffee bean extract (GCBE). We systematically examined potential evidence for conjugation in GCBE. Firstly, we analyzed changes in key components, such as phenolic compounds, sugars, and proteins. Secondly, we assessed the impact on GCBE’s biological properties, including antioxidant activity, antimicrobial effects, and toxicity. Finally, we combined the results and discussed the alterations observed in protein size and FT-IR to establish conclusive evidence of conjugation resulting from plasma treatment.

### 3.1. Changes in GCBE Composition

The sugar profile analysis by HPLC revealed that only sucrose was present in the GCBE samples. The sucrose levels remained relatively unchanged following plasma treatment, suggesting its stability. Sucrose is a non-reducing sugar and lacks functional groups susceptible to plasma-induced chemical reactions [23,24]. Therefore, it is unsurprising that plasma treatment did not significantly affect the sucrose levels in the GCBE.

On the other hand, the total free amino acid content decreased with increasing plasma treatment time, and the 17 amino acids showed varying degrees of change. Several amino acids showed no significant changes with plasma treatment, including aspartate, threonine, serine, glutamic acid, proline, alanine, and cysteine. This suggests that these amino acids are relatively stable and not susceptible to oxidation by the reactive oxygen species (ROS) generated during plasma treatment. In contrast, higher plasma treatment times resulted in reduced valine, histidine, and lysine levels, which contain reactive functional groups prone to oxidation. The decrease in the levels of these amino acids can be attributed to the oxidative reactions initiated by ROS during plasma treatment. Conversely, slight increases were observed in glycine, methionine, isoleucine, leucine, tyrosine, phenylalanine, and arginine levels. These increases might be attributed to protein fragmentation or the formation of reactive intermediates during plasma treatment that react with these amino acids to form adducts or conjugates [25].

The total phenolic content (TPC) increased at P10 and P35 but decreased at P60. The increase in TPC at P10 and P35 could be attributed to the breakdown of more significant phenolic compounds into smaller ones through plasma treatment, leading to an increase in the TPC [11]. At the same time, the decrease in TPC at P60 suggests that further plasma treatment led to the degradation of the phenolic compounds. However, the phenolic profile of the GCBE showed that chlorogenic acid and caffeine, the two most abundant antioxidants in GCBEs [26], decreased significantly at P35. The reduction in chlorogenic acid and caffeine levels might be due to degradation [27]. 

The discrepancy between the results obtained from the Folin–Ciocalteu method and HPLC analysis of phenolic compounds post-plasma treatment underscores the need for a closer examination of potential underlying mechanisms. The observed augmentation in TPC suggests the possibility of an accumulation of phenolic compounds after plasma treatment [11,15]. However, the decrease in other antioxidants, as revealed by HPLC, implies that these compounds may not contribute to the observed TPC increase. This intriguing disparity hints at the possibility that the phenolic compounds present may not have conventional antioxidant behaviors or may undergo transformations that affect their reactivity. Phenolic compounds, renowned for their dynamism, can undergo processes such as oxidation and polymerization, potentially giving rise to new phenolic compounds endowed with enhanced reducing properties [28].

Furthermore, the alterations in the amino acid profile, characterized by increases in specific amino acids including glycine, methionine, isoleucine, leucine, tyrosine, phenylalanine, and arginine, may also exert an influence on the TPC. Of particular interest are tyrosine and phenylalanine, which contain aromatic rings and functional groups that have the potential to interact with phenolic compounds through redox reactions, culminating in the formation of new compounds harboring antioxidant properties. Remarkably, previous research by Hwang et al. [29] demonstrated the conjugation of chlorogenic acids with glutamic acid, yielding a compound with augmented antioxidant and antiviral activities.

Intriguingly, the protein size variation could contribute to the elevation in TPC. The SDS-PAGE analysis unveiled a reduction in smaller proteins alongside an increase in larger ones, suggesting a polymerization process and potential conjugation of proteins. Conjugation involves the linkage of compounds through non-covalent or covalent bonding, and protein conjugates often exhibit enhanced bioactivity, including bolstered antioxidant, antimicrobial, and anticancer activities [30,31,32]. Plasma treatment may augment the conjugation between phenolic compounds and proteins, potentially leading to the reduced detection of free phenolic compounds by HPLC. It is worth noting that the Folin–Ciocalteu method utilized for measuring the TPC does not differentiate between distinct phenolic compounds and may also detect conjugated ones, thereby explaining the observed increase in TPC.

Collectively, these intricate interactions involving phenolic compounds, amino acids, and proteins offer a plausible explanation for the heightened TPC despite the concurrent decrease in other antioxidants as quantified by HPLC. This multifaceted interplay underscores the complexity of the mechanisms at play during plasma treatment and invites further investigation into the underlying processes.

### 3.2. Changes in GCBE Bioactivities

A further evaluation of the antioxidant activity using different tests revealed exciting findings. The plasma treatment had different effects on the antioxidant activity of the GCBE, depending on the test used. The DPPH assay showed that plasma treatment improved the antioxidant activity of the GCBE. However, the ABTS (increasing trend) and FRAP (reducing trend) assays did not show significant differences, which suggests that plasma treatment may not significantly impact the antioxidant capacity of the GCBE as measured by these assays. The cellular oxidative stress status assay showed that low-dose GCBE increased the ROS intensity in cells, which is unsurprising as many natural antioxidants can initially induce ROS production in cells.

Interestingly, at higher concentrations, the GCBE exhibited antioxidant activity with concentration dependence, which suggests that it may have a hormetic effect on cells. This hormetic effect is characterized by a biphasic dose-response relationship, where a low dose of a stressor (in this case, the GCBE) can stimulate a beneficial response in cells, while high doses can have a negative effect [33]. The potential hormetic effect of GCBE on cells is an area that warrants further investigation.

According one review [34], there are many antioxidant mechanisms for antioxidants. The results suggest that the plasma treatment improved the ability of GCBE to scavenge free radicals (DPPH and ABTS assays) through hydrogen atom transfer (HAT) or electron transfer mechanisms (SET). However, it did not affect the reducing power (FRAP assay). The lack of significant changes in the FRAP assays indicates that plasma treatment may not affect the SET antioxidant mechanism of the GCBE and might only improve the HAT antioxidant mechanism.

In simpler terms, the plasma treatment made GCBE better at neutralizing harmful free radicals in specific tests, but it did not improve its ability to donate electrons to ferric ions or its cellular antioxidant activity. These diverse effects show that the plasma treatment can change the bioactive compounds of the GCBE, making some parts more effective at scavenging radicals while altering other parts. It is also possible that there was a generation of new reactive intermediates or compounds during plasma treatment, which could contribute to the increased antioxidant activity [17,34]. Furthermore, the decrease in cellular protection observed in the CAA assay raises concerns about the intracellular behavior of plasma treatment on GCBE. It suggests that the processing technique might have altered the accessibility or bioavailability of active antioxidant compounds, leading to diminished cellular protection.

For antimicrobial activity, conjugation could explain the slight trend in enhanced antimicrobial activity of the GCBE after plasma treatment [32]. The presence of an inhibition zone around P35 suggests that the plasma-treated GCBE can somewhat negatively affect the growth of *S. aureus*. To explain this observation, we can consider several potential factors. Firstly, the plasma treatment might effectively inhibit the growth of most *S. aureus* cells, but some cells could be less susceptible, forming small colonies within the zone. Secondly, *S. aureus* is known for its heteroresistance, where specific subpopulations have inherent resistance to antimicrobial agents, and these resistant subpopulations might be responsible for the presence of small colonies. Lastly, slow-growing subpopulations within *S. aureus* could contribute to the growth of colonies within the inhibition zone over a more extended period [35].

### 3.3. Evidence of Conjugation

So far, the combined results underscore the profound impact of cold plasma treatment on GCBEs’ chemical composition and bioactivity. The observed enhancements in antimicrobial activity, radical scavenging capacity, and protein size following plasma treatment suggest potential modifications to the bioactive compounds within the extract. It is evident that plasma treatment can induce transformative changes in GCBEs, with shorter exposures predominantly amplifying antioxidant activity and total phenolic content, while prolonged exposure tends to result in reductions in both. These dynamic alterations appear to stem from two competing mechanisms induced by plasma treatment. On one hand, plasma may lead to a reduction in bioactive compounds, while on the other, it enhances them. Consequently, at shorter exposure times, the enhancement appears to have a more significant impact than the loss, while at prolonged exposure, the loss tends to prevail.

The observed trend of loss of bioactive compounds and antioxidant potential with increased processing time in plasma processing, as evidenced in various food products [36,37], is congruent with the findings presented here. The decline in total phenols is attributed to the reaction between phenolic compounds and reactive oxygen species.

Interestingly, similar to this study’s results, an increase in antioxidant activity upon plasma processing has been observed in camu camu juice [38] and green tea leaves [11]. It is suggested that this increase may be due to the depolymerization of cell wall polysaccharides during plasma treatment, facilitating better extraction of conjugated phenolic compounds. However, as previously mentioned, the heightened TPC despite the concurrent decrease in other antioxidants quantified by HPLC and the increase in protein size suggest the involvement of conjugation processes.

Various synthetic methods are employed to create phenolic-protein conjugates with distinct properties. The alkaline method involves the oxidation of phenolic compounds at a pH of 9 using NaOH, leading to the formation of quinones and subsequent dimerization into more reactive dimer quinones. These quinones can react with nucleophilic side chains of proteins, such as those in lysine and tryptophan, forming EGCG-protein conjugates through Schiff bases and Michael-type adducts [39]. The free radical-mediated grafting method relies on redox pairs of hydrogen peroxide and ascorbic acid to generate radicals that induce protein radicals, which then covalently link with phenolic compounds [40]. Enzyme-catalyzed methods employ polyphenol oxidases to convert phenolic compounds into electrophilic quinones that react efficiently with nucleophilic amino groups of proteins, forming Schiff bases and Michael-type adducts [41]. Lastly, the chemical coupling method employs chemical coupling reagents such as glutaraldehyde to facilitate the conjugation of phenolic compounds with proteins, enhancing various properties such as antioxidant activity, stability, and emulsifying properties of the proteins [42]. These diverse methods offer flexibility in designing phenolic–protein conjugates for specific applications.

The primary conjugation mechanism in this study may involve free radical grafting methods facilitated by the radicals produced during plasma treatment. However, instead of relying on the redox pair reaction of hydrogen peroxide and ascorbic acid, radicals from plasma, such as ozone, hydrogen peroxide, and nitric oxide, could induce macroprotein radicals, thus initiating the conjugation process. 

The FT-IR analysis further furnished valuable insights into alterations in alkene, ether, carbonyl, and hydroxyl groups, corroborating the hypothesis of phenolic compound and protein interactions. Thus, the formation of protein-phenolic conjugates likely contributes to the observed increase in protein intensity on SDS-PAGE.

The decline in free amino acids and phenolic compounds following plasma treatment underscores the dynamic interplay between these compounds during treatment. To unravel the specific mechanisms driving these changes, further investigations, including protein identification, structural analysis, and phenolic compound characterization, are imperative.

It is important to note that our study has certain limitations. While we have gained valuable insights into the effects of direct plasma treatment on the GCBE, we recognize that this approach may not provide a complete understanding of the potential benefits associated with GCBE production. Plasma treatment could potentially enhance both bioactivity and extraction efficiency if applied before or during the extraction process. Future investigations focusing on the pre-extraction or in situ application of cold plasma may offer a more comprehensive evaluation of its impact on bioactive compounds and extraction efficiency. These avenues of research hold promise for further optimizing plant extract production while addressing the constraints of our current study.

## 4. Materials and Methods

### 4.1. Materials and Chemicals

This study used green coffee beans of *Coffea arabica* obtained from the Royal Project Foundation in Thailand, cultivated in the country’s northern region, and processed using the standard wet method. They were vacuum sealed, packed in an aluminum foil bag, and kept at −18 °C until further use.

Four sugar standards (sucrose (99.5%), glucose (99.5%), fructose (99%), and galactose (99%)) and eighteen phenolic compound standards (gallic acid (99%), protocatechuic acid (98%), *p*-hydroxybenzoic acid (98%), catechin (97%), chlorogenic acid (5-caffeoylquinic acid (5-CQA), 99.5%), caffeine (99%), vanillic acid (97%), caffeic acid (98%), syringic acid (95%), epicatechin (98%), vanillin (98%), *p*-coumaric acid (98%), ferulic acid (99%), sinapic acid (99%), rutin (95%), myricetin (96%), quercetin (95%), and trans-cinnamic acid (98%)), 2,2-diphenyl-1-picrylhydrazyl (DPPH), 2,2′-azino-bis(3-ethylbenzothiazoline-6-sulfonic acid) diammonium salt (ABTS), Trolox (97%), 2,4,6-tri(2-pyridyl)-s-triazine (TPTZ, 98%), ferrous sulfate heptahydrate (99.5%), 3-(4,5-dimethylthiazol-2-yl)-2,5-diphenyl tetrazolium bromide (MTT), and diacetyldichlorofluorescein (DCFH-DA, 95%)) were purchased from Merck, Darmstadt, Germany. Seventeen standard amino acids (aspartic acid, threonine, serine, glutamic acid, proline, glycine, alanine, cysteine, valine, methionine, isoleucine, leucine, tyrosine, phenylalanine, histidine, lysine, and arginine) were purchased from FUJIFILM Wako Pure Chemical Corporation, Osaka, Japan. HPLC-grade methanol and acetonitrile were purchased from RCI Labscan, Bangkok, Thailand. All solutions and dilutions were made using deionized water generated using a Milli-Q water system (Zeneer up 900, Seoul, Republic of Korea). Laemmli sample buffer, Coomassie G-250 staining solution, and protein markers (2 to 250 kDa) were obtained from Bio-Rad Laboratories, Inc., Hercules, CA, USA. All the other chemicals used were analytical grade.

### 4.2. Preparation of Green Coffee Bean Extract

The green coffee beans were extracted using ultrasonication, following a method adapted from Iftikhar et al. [43]. The green coffee beans, with a moisture content of 8–9%, were ground into a powder using a hammer mill with a 1 mm mesh. GCBE was extracted using a 37 kHz ultrasonicator (Model Elma S 100 H, Elma Schmidbauer GmbH, Singen, Germany) with a solid-to-liquid ratio of 1:4 (*w*/*w*) for 30 min, using water as the extraction solvent. The resulting mixture was then passed through a 10 µm polypropylene sieve bag to remove any solid particles. The resulting filtrate was adjusted to pH 6.6 using 0.1 M NaOH. It was used as the GCBE (control) for the subsequent treatment.

### 4.3. Cold Plasma Treatment

**Figure A1:**
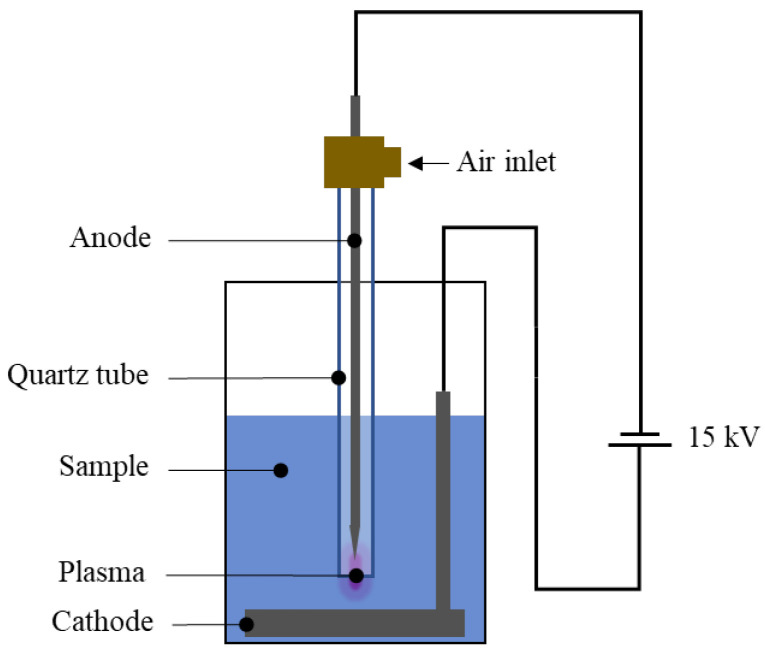
Schematic of the underwater plasma jet system.

**Figure A2:**
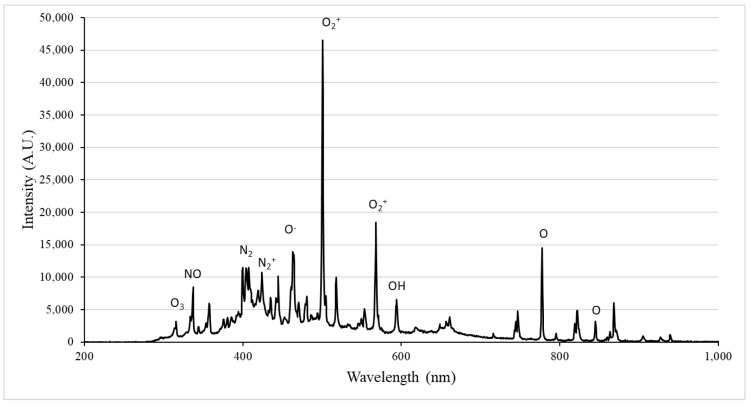
Optical emission spectrum of underwater plasma jet showing the generated radicals.

The study utilized an underwater plasma jet system. A 125 W 15 kV neon transformer powered the plasma system. The electrode system consisted of a pen-shaped stainless-steel electrode placed inside a quartz tube with a 5 mm inner diameter, with the electrode tip positioned 3 mm above the jet nozzle. The system is illustrated in Figure A1. An atmospheric cold plasma jet was generated by blowing 0.05 L/min of dehumidified air through the quartz tube. The plasma system was enclosed in a controlled chamber, with the temperature and humidity maintained at 25 ± 3 °C and 60 ± 10% RH, respectively. The radical profile generated by the system is shown in Figure A2.

During the experiment, 70 mL of the GCBE (control) in a beaker was exposed to plasma for 10 min (P10), 35 min (P35), and 60 min (P60). Each treatment was performed in triplicate.

### 4.4. Total Polyphenolic Content (TPC)

The method to measure total phenolic content was adapted from Upadhyay et al. [44]. A 1 mL sample was mixed with 7.5 mL of a saturated sodium carbonate solution and 0.5 mL of Folin–Ciocalteu phenol reagent. Distilled water was added to the mixture up to a final volume of 10 mL and vortexed for 30 s. The absorbance was measured at 760 nm using a UV-vis spectrometer (UV1800, Shimadzu, Kyoto, Japan), and a standard curve was used to determine the total phenolic compound content in g gallic acid equivalent per L (g GA/L) units. Each sample was measured in triplicate for accuracy.

### 4.5. Sugar Profile

Four sugars, including sucrose, glucose, and fructose, were analyzed using the Shodex™ Capture the Essence method developed by Yamaguchi et al. [45], a high-performance liquid chromatography system (Shimadzu, Kyoto, Japan), and a Shodex HILICpak VG-50 4E column (4.6 mm inner diameter × 250 mm length, Resonac Holdings Corporation, Tokyo, Japan), as well as a refractometer (Shimadzu, Kyoto, Japan). The eluent comprised acetonitrile, methanol, and water (85:10:5, *v*/*v*), which flowed at rate of 0.6 mL/min. The temperature was maintained at 50 °C using a CTO-20AC column oven (Shimadzu, Kyoto, Japan). The sample was prepared by diluting the extract 20-fold with acetonitrile (50:50, *v*/*v*) and filtering it through a 0.20 μm membrane filter. A 10 μL aliquot was injected for each run, and the sample was measured in duplicate.

### 4.6. Free Amino Acid Profile

A high-performance liquid chromatography system (Shimadzu, Kyoto, Japan) with post-column reaction was used to determine the concentrations of seventeen amino acids according to the method of Yoshida et al. [46], with some modification. A Shim-pack Amino-Na column (100 mm × 6.0 mm inner diameter, 5 µm; P/N: 228-18837-91, Shimadzu, Kyoto, Japan) was used to separate the amino acids. The mobile phase consisted of three types: A, B, and C. Mobile phases A and B were sodium citrate buffers with pH values of 3.23 and 10.0, respectively. A 0.2 M sodium hydroxide solution was used as mobile phase C. *o*-phthalaldehyde (OPA) and N-acetylcysteine were used as reaction reagents, resulting in the detection of fluorescent derivatives. The column oven temperature was maintained at 60 °C, the flow rate was 0.4 mL/min, the running time was about 70 min, the injection volume was 10 μL, and a fluorescence detector was used. The total free amino acid concentration was calculated as the sum of all identified amino acids. Each sample was measured in duplicate.

### 4.7. Chlorogenic Acid and Caffeine Contents

Chlorogenic acid and caffeine contents were analyzed using a high-performance liquid chromatography instrument (1260 Infinity II LC system) equipped with an autosampler and diode array detector (Agilent Technology, Santa Clara, CA, USA). The method was adapted from Awwad et al. [1]. The sample was filtered with a 0.45 µm filter membrane before 10 µL of the sample was injected into the system. Agilent Eclipse Plus C18, 4.6 mm × 150 mm, (5 µm), was used for chromatographic separation. Mobile phase A was 0.1% trifluoroacetic acid, and mobile phase B was acetonitrile. Chlorogenic acids and caffeine were separated by gradient as follows: 0–20 min A (90%–80%) B (10%–20%); 20–30 min A (80%–80%) B (20%–20%); 30–35 min A (80%–95%) B (20%–5%); and 35–40 min A (95–95%) B (5–5%) at a flow rate of 1.5 mL/min. The sample was measured at 254 nm and compared to chlorogenic acid (5-caffeoylquinic acid (5-CQA)) and caffeine standards. The sample was measured in duplicate.

### 4.8. Phenolic Profile

The sample was analyzed using a high-performance liquid chromatography system (Shimadzu, Kyoto, Japan), The method used for chromatographic separation was adapted from Liaudanskas et al. [47]. Before injection into the system, the sample was filtered with a 0.45 µm filter membrane. Inersil C18 (250 × 4.6 mm) GL Sciences Inc., Tokyo, Japan, was used for chromatographic separation. Mobile phase A was 2% acetic acid in water, and mobile phase B was acetonitrile.

The gradient used for the separation of phenolic compounds was as follows: 0–5 min A (95%–93%) B (5%–7%); 5–25 min A (93%–92%) B (7%–8%); 25–27 min A (92%–89%) B (8%–11%); 27–32 min A (89%–88%) B (11%–12%); 32–44 min A (88%–85%) B (12%–15%); 44–52 min A (85%–85%) B (15%–15%); 52–53 min A (85%–68%) B (15%–32%); 53–59 min A (68%–68%) B (32%–32%); 59–63 min A (68%–80%) B (32%–20%); 63–69 min A (80%–10%) B (20%–90%); 69–74 min A (10%–10%) B (90%–90%); 74–75 min A (10%–95%) B (90%–5%); and 75–85 min A (95–95%) B (5–5%) at a flow rate of 1 mL/min.

The sample was measured at 280 nm and compared to 17 phenolic compounds: gallic acid, protocatechuic acid, *p*-hydroxybenzoic acid, catechin, chlorogenic acid (5-caffeoylquinic acid (5-CQA)), caffeine, vanillic acid, caffeic acid, syringic acid, epicatechin, vanillin, *p*-coumaric acid, ferulic acid, sinapic acid, rutin, myricetin, quercetin, and trans-cinnamic acid. The sample was measured in duplicate.

### 4.9. Evaluation of Antioxidant Activity

#### 4.9.1. DPPH Free Radical-Scavenging Activity Determination

The DPPH free-radical scavenging assay used in this study was adapted from Siripatrawan and Harte [48]. To conduct the assay, 1 mL of the sample was added to 2 mL of 0.2 mM 2,2-diphenyl-1-picrylhydrazyl (DPPH) in 80% methanol. The mixture was then vigorously mixed and kept in the dark at room temperature for 30 min. The absorbance at 517 nm was measured using a UV-vis spectrophotometer (UV1800; Shimadzu, Kyoto, Japan). A blank was prepared using distilled water instead of the sample. Gallic acid was used to construct a standard curve. The antioxidant activity was expressed as g gallic acid equivalent per L of the extract (g GAE/L). The sample was measured in triplicate.

#### 4.9.2. ABTS Radical Scavenging Assay

The antioxidant activity of the samples was determined using the ABTS method adapted from Re et al. [49]. A radical solution was prepared by mixing 2.45 mM of potassium persulfate and 7 mM of 2,2′-azino-bis(3-ethylbenzothiazoline-6-sulfonic acid) diammonium salt (ABTS) solution in 20 mM sodium acetate buffer (pH 4.5). The solution was incubated in the dark at room temperature for 12–16 h to obtain a stable and dark blue-green radical solution. A working solution was prepared by diluting the radical solution with 95% ethanol to an absorbance of 0.70 ± 0.02 at 734 nm. Next, 20 μL of sample solution was added to 2 mL of the working solution and incubated at room temperature in the dark for 6 min. The absorbance was measured at 734 nm, and the ABTS radical-scavenging activity was calculated against a standard curve of Trolox (0.1–1.0 g/L). The results are expressed as g Trolox equivalent per L of the extract (g TE/L), and the sample was measured in triplicate.

#### 4.9.3. Ferric Reducing Antioxidant Power (FRAP) Assay

The FRAP assay used in this study was adapted from Benzie and Strain [50]. The FRAP reagent solution was prepared by mixing 2.5 mL of 10 mM 2,4,6-tri(2-pyridyl)-s-triazine (TPTZ) solution in 40 mM HCl, 2.5 mL of 20 mM ferric chloride hexahydrate solution, and 20 mL of 300 mM acetate buffer (pH 3.6) and incubated at 37 °C for 30 min to obtain the FRAP solution. A 50 μL volume of the sample was added to 750 μL of the FRAP solution and kept in the dark for 30 min. The change in the solution color was measured at 593 nm using a spectrophotometer. A standard curve was prepared using ferrous sulfate heptahydrate, and the results were expressed as g ferrous sulfate equivalent per L of the extract (g Fe^2+^/L). The sample was measured in triplicate.

#### 4.9.4. Cellular Antioxidant Activity (CAA) Assay

The HepG2 cell line (CLS Cell line service GmbH, Eppelheim, Germany) was studied using the cellular antioxidant activity (CAA) assay adapted from Wolfe and Liu [51]. The cells were transferred to a 96-well plate at an initial concentration of 1 × 10^4^ cells/well. Then, they were incubated at 37 °C inside a CO_2_ incubator for 24 h. Nutrient media and the GCBE at various concentrations (6.3–50 mg/L) were added at a volume of 100 µL and incubated at 37 °C in a CO_2_ incubator for 24 h. The treated cells were washed with phosphate-buffered saline (PBS) and incubated with the MTT reagent (5 g/L) for 4 h. Next, 10 µM diacetyl dichlorofluorescein (DCFH-DA) was added and the plate was incubated at 5% CO_2_ and 37 °C for 30 min. The cells were rewashed with PBS, and then induced with 125 µM hydrogen peroxide (H_2_O_2_) and incubated at 5% CO_2_ and 37 °C for 30 min. The test was performed along with three control samples: (1) basal control, where no GCBE was added to the cells and no H_2_O_2_ induction; (2) positive control, where no GCBE was added; and (3) negative control, where 50 mg/L Trolox was added to the cells instead of the GCBE. To assess the compounds’ antioxidant capacity, the samples’ cellular fluorescence, measured using a microplate reader (BioTek Synergy H4 Hybrid Reader, BioTek Instruments Inc., Winooski, VT, USA), was compared to that of the positive control as a percentage. The percentage decrease in cellular fluorescence, relative to the positive control, indicates the antioxidant capacity of the compounds. The sample was measured in triplicate.

### 4.10. Sodium Dodecyl Sulfate-Polyacrylamide Gel Electrophoresis (SDS-PAGE)

SDS-PAGE was performed according to Laemmli [52]. SDS-PAGE was accomplished using a 12% acrylamide running gel at pH 8.8 and a 4% loading gel at pH 6.8. The complex/macro molecules were determined by preparing the samples with a 2-fold dilution in 2× Laemmli sample buffer containing 65.8 mM Tris-HCl (pH 6.8), 26.3% (*w*/*v*) glycerol, 2.1% SDS, 0.01% bromophenol blue, and 2.5% (*v*/*v*) 2-mercaptoethanol. The extract was boiled for 3 min before loading (20 μL) into the gel. After the electrophoresis, the acrylamide gel was fixed in 40% methanol and 10% acetic acid for 30 min. The gel was then washed in deionized water and stained using a Coomassie G-250 staining solution (Bio-Rad, Hercules, CA, USA) and then incubated in a de-staining solution (25% methanol and 7% acetic acid) overnight before scanning. Protein markers with multiple molecular weights from 2 to 250 kDa (Bio-Rad, Hercules, CA, USA) were used in this analysis.

### 4.11. Fourier-Transform Infrared (FT-IR) Spectroscopy 

GCBE samples were lyophilized using a freeze-drying machine (Harvest Right™, Salt Lake City, UT, USA). The samples in powder form were pressed with KBr powder into a pellet for FT-IR analysis. The spectra were recorded in the 4000–400 cm^−1^ range at a 4 cm^−1^ resolution using an FT-IR spectrophotometer (Frontier, PerkinElmer, Waltham, MA, USA). 

### 4.12. Antimicrobial Testing

The antimicrobial activity test was based on the disk diffusion method described in the Manual of Clinical Microbiology [53], with some modifications. *Staphylococcus aureus* subsp. *aureus* ATCC 25923 (*S. aureus*) was used for the test. *S. aureus* suspensions at a 10^6^ CFU/mL concentration were streaked onto Mueller Hinton agar plates, and sterile Whatman filter paper No. 1 discs (6 mm diameter) containing 20 µL of the sample were placed on the inoculated surface. The plates were then incubated at 37 °C for 24 h, and the inhibition zones were measured using a ruler. The test was performed with 10% DMSO as the negative control and 1 g/L gentamycin as the positive control.

### 4.13. Cytotoxicity

Cytotoxicity was assessed using the MTT assay method described by Radapong et al. [54]. The HepG2 cell line (CLS Cell line service GmbH, Eppelheim, Germany) was used in the analysis. The cells were seeded in 96-well culture plates at a density of 1 × 10^4^ cells/well and treated with varying concentrations of GCBE (0–400 μg/mL) for 24 and 48 h. After treatment, the cells were washed with PBS and incubated with the MTT reagent (5 g/L) for 4 h. The blue-colored formazan product was extracted with 0.1 mL dimethyl sulfoxide (DMSO), which was monitored at 540 and 630 nm using a microplate reader (BioTek Synergy H4 Hybrid Reader, BioTek Instruments Inc., Winooski, VT, USA). Cell viability was expressed as a percentage of untreated cells (100% viability). The experiment was conducted in triplicate.

### 4.14. Statistical Analysis

Statistical analyses were conducted using SPSS version 17.0 (SPSS Inc., Chicago, IL, USA). The results are presented as mean ± standard deviation (SD). One-way analysis of variance (ANOVA) was performed to compare data among the plasma-treated samples (P10, P35, and P60). Duncan’s Multiple Range Test (MRT) was used to determine significant differences between samples at a significance level of *p*-value ≤ 0.05. Student *t*-test was used to compare the samples with the control at a significance level of *p*-value ≤ 0.05.

## 5. Conclusions

In summary, this study has unveiled the intricate impact of cold plasma treatment on green coffee bean extracts (GCBEs), elucidating complex changes in their chemical composition and bioactivity. The reductions in key antioxidants, specifically chlorogenic acid and caffeine, the augmentation of protein size, and FT-IR changes suggest potential protein–phenolic conjugation. Furthermore, our research demonstrates that short plasma exposure can significantly enhance antioxidant activity and total phenolic content (TPC). This results in a noteworthy 66% increase in radical scavenging activity after 35 min of plasma treatment. However, it is essential to note that prolonged exposure diminishes these benefits, highlighting the critical role of treatment duration. These intricate transformations indicate the complexity of plasma-induced modifications. These findings offer valuable insights for the food industry and nutraceutical research, presenting innovative approaches to optimizing the production processes of green coffee bean extracts and potentially other plant extracts. This investigation lays the foundation for fully leveraging the benefits of cold plasma treatment to enhance the bioactivity and functionality of plant extracts, benefiting both the food industry and health-conscious consumers seeking more potent and healthier natural products. Further investigations are warranted to elucidate the mechanisms underlying the observed effects and fully harness the benefits of cold plasma treatment for producing higher quality and more bioactive natural products.

## Figures and Tables

**Table 1:** Total free amino acids, sucrose, total phenolic content, chlorogenic acid, and caffeine of GCBE (control) and GCBEs treated with plasma for 10 min (P10), 35 min (P35), and 60 min (P60).

Measurement	Control	Treatment		
		P10	P35	P60
Total free amino acids (mg/L)	392.16 ± 1.62	387.21 ± 1.47 ^a,^*	335.71 ± 39.19 ^b^	316.00 ± 15.64 ^b,^*
Sucrose (g/L)	23.50 ± 1.27	23.06 ± 2.58	23.93 ± 1.11	23.91 ± 1.63
Total phenolic content (g GAE/L)	5.27 ± 0.16	6.05 ± 0.54 ^b^	7.63 ± 0.26 ^a,^*	6.75 ± 0.28 ^b,^*
Chlorogenic acid (g/L)	5.10 ± 0.01	4.84 ± 0.62 ^a,^*	4.75 ± 0.48 ^a,^*	4.17 ± 0.77 ^b,^*
Caffeine (g/L)	1.54 ± 0.03	1.48 ± 0.02 *	1.48 ± 0.04	1.53 ± 0.04

^a,b^ Values with different letters within the same row differ significantly with a *p*-value ≤ 0.05, as indicated by Duncan’s new multiple range test. * Values differ significantly from the control with a *p*-value ≤ 0.05, as indicated by Student’s *t*-test.

**Table 2:** Free amino acid profile of GCBE (control) and GCBEs treated with plasma for 10 min (P10), 35 min (P35), and 60 min (P60).

Amino Acid (mg/L)	Control	Treatment		
		P10	P35	P60
Aspartate	22.90 ± 0.64	22.71 ± 0.65	21.58 ± 3.00	22.55 ± 2.09
Threonine	14.78 ± 0.08	14.58 ± 0.20 *	14.45 ± 1.55	14.41 ± 1.45
Serine	10.71 ± 0.12	10.54 ± 0.06 *	10.25 ± 1.08	10.02 ± 0.87
Glutamic acid	86.37 ± 0.83	85.09 ± 0.77 *	90.54 ± 13.10	92.67 ± 11.15
Proline	15.32 ± 0.08	15.55 ± 0.12 *	21.33 ± 7.80	21.56 ± 5.55
Glycine	2.79 ± 0.13	2.60 ± 0.09 ^b,^*	3.09 ± 0.25 ^a^	3.20 ± 0.14 ^a,^*
Alanine and cysteine	54.42 ± 0.50	54.01 ± 0.51	57.93 ± 5.47	60.42 ± 3.72
Valine	40.18 ± 0.75	39.96 ± 0.17 ^a,^*	32.96 ± 5.68 ^ab^	29.73 ± 1.14 ^b,^*
Methionine	ND	ND	9.81 ± 7.65 ^ab^	13.77 ± 1.17 ^a,^*
Isoleucine	2.74 ± 0.05	2.78 ± 0.04 ^b^	5.98 ± 2.43 ^a^	7.54 ± 0.14 ^a,^*
Leucine	5.03 ± 0.47	5.45 ± 0.07 ^b,^*	6.58 ± 1.07 ^ab^	7.25 ± 0.27 ^a,^*
Tyrosine	ND	ND	1.99 ± 1.55 ^ab^	3.12 ± 0.16 ^a,^*
Phenylalanine	3.66 ± 0.02	3.47 ± 0.05b *	8.37 ± 3.75 ^a^	10.94 ± 0.87 ^a,^*
Histidine	21.12 ± 3.43	20.16 ± 2.37 ^a^	8.98 ± 9.94 ^ab^	2.57 ± 0.28 ^b,^*
Lysine	35.52 ± 0.18	34.97 ± 0.29 ^a,^*	20.64 ± 8.21 ^b^	16.26 ± 1.14 ^b,^*
Arginine	76.62 ± 2.13	75.33 ± 1.76 ^a^	ND	ND

^a,b^ Values with different letters within the same row differ significantly with a *p*-value ≤ 0.05, as indicated by Duncan’s new multiple range test. * Values differ significantly from the control with a *p*-value ≤ 0.05, as indicated by Student’s *t*-test. ND = not detected.

**Table 3:** Phenolic profile of GCBE (control) and GCBE treated with plasma for 35 min (P35).

Phenolic Compound (mg/L)	Control	P35	*p*-Value
Chlorogenic acid	6033.93 ± 82.96	5585.13 ± 118.27	0.0481 *
Caffeine	1466.18 ± 21.87	1365.64 ± 23.50	0.0474 *
Vanillic acid	995.86 ± 13.49	945.83 ± 65.72	0.4022
Vanillin	32.02 ± 3.45	29.98 ± 2.66	0.5753
*p*-Coumaric acid	48.65 ± 5.31	45.01 ± 6.63	0.9591
Myricetin	148.01 ± 43.95	148.58 ± 3.44	0.9870

* Values differ significantly with a *p*-value ≤ 0.05, as indicated by Student’s *t*-test.

**Table 4:** ABTS radical scavenging activity and FRAP assay results of GCBE (control) and GCBEs treated with plasma for 35 min (P35).

Measurement	Control	P35	*p*-Value
ABTS (g TE/L)	2.89 ± 0.04	2.96 ± 0.10	0.3046
FRAP (g Fe^2+^/L)	30.19 ± 1.68	28.71 ± 0.97	0.2568

## Data Availability

All data are contained within the article.

## Appendix A

The plasma systems are illustrated in Figure A1. The electrical energy transferred to the plasma is responsible for the excitation of several chemical species. To see the excited radicals, optical emission spectroscopy was used. Figure A2 shows the radical profile of the underwater plasma jet used in the study. The detected radicals include O, O^•^, O_2_^+^, O_3_, OH^•^, NO, N_2_, and N_2_^+^. These were the radicals that could react with the GCBE.
